# Diagnosis and clinical course of ocular ischemic syndrome with retinal vascular abnormalities due to unilateral ocular artery and internal carotid artery stenosis in a child with neurofibromatosis type 1: a case report

**DOI:** 10.1186/s12886-020-01670-z

**Published:** 2020-10-23

**Authors:** Hiroaki Sakai, Kosuke Kawata, Jun Masuoka, Tomohisa Nishimura, Hiroshi Enaida

**Affiliations:** 1 Department of Ophthalmology, Faculty of Medicine, Saga University, 5-1-1 Nabeshima, Saga, 849-8501 Japan; 2 Department of Neurosurgery, Faculty of Medicine, Saga University, 5-1-1 Nabeshima, Saga, 849-8501 Japan; 3 Mikawa Eye Clinic, 4-3-21 Matsubara, Saga, 840-0831 Japan

**Keywords:** Neurofibromatosis 1, Ophthalmic artery stenosis, Internal carotid artery stenosis, Ocular ischemic syndrome, Retinal vascular abnormalities, Neovascular glaucoma, Multimodal imaging

## Abstract

**Background:**

Neurofibromatosis type 1 (NF1) is a hereditary disease that causes neurofibromas generally, but it has been reported to sometimes be associated with various forms of blood vessel stenosis, occlusion and vascular abnormalities of unknown mechanism. However, a symptomatic case with simultaneous ophthalmic artery stenosis and internal carotid artery stenosis is an extremely rare pathogenesis in a child with NF1. In this report, we performed the diagnosis and observation using various imaging modalities for this rare pediatric case.

**Case presentation:**

A 6-year-old girl diagnosed with NF1 presented with gradual visual loss in the right eye. Best corrected visual acuity (BCVA) was 20/40 OD and the intraocular pressure (IOP) was normal in both eyes. Retinal vascular abnormalities with tortuous vessels and optic disc pallor were observed in the right fundus. Widefield fluorescein angiography revealed multiple sites of neovascularization and a large non-perfusion area in the peripheral retina. Optical coherence tomography angiography showed retinal vascular abnormalities in the right eye and revealed differences in inner retinal thickness and blood flow signal between the left and right eyes. Laser speckle flowgraphy showed that chorioretinal blood flow was significantly decreased in the right eye. Cerebral angiography revealed the right ophthalmic artery was significantly narrowed throughout. In addition, Magnetic resonance angiography revealed that the right internal carotid artery was significantly narrowed in the ophthalmic segment. We diagnosed ophthalmic artery and internal carotid artery stenosis with retinal vascular abnormalities and ocular ischemic syndrome in NF1. Because IOP increased to 35 mmHg, due to neovascular glaucoma in addition to mild vitreous hemorrhage occurred, panretinal photocoagulation was performed after intravitreal bevacizumab injection. After treatments, IOP normalized, but BCVA decreased to 20/100 OD. Arterial spin labeling showed normal cerebral blood flow. The patient is currently being carefully monitored.

**Conclusions:**

We have described the diagnosis and treatment of ocular ischemic syndrome due to multiple arteries stenosis in a child with NF 1. Utilization of various imaging modalities was helpful in diagnosing the complicated pathogenesis. However, since direct intervention by neurosurgery is not possible in this case, it is expected that treatment will be extremely difficult in the future.

## Background

Neurofibromatosis type 1 (NF1) is a hereditary disease that causes neurofibromas throughout the body [1], but NF1 is sometimes also accompanied by unexplained stenosis, occlusion and abnormalities of various blood vessels [2–8]. There have been reports of serious systemic complications due to stenosis of the renal artery or internal carotid artery, even in childhood [2–7]. On the one hand, there have been only two reports of ophthalmic artery occlusion with intracranial lesions that developed in NF1; detailed imaging, diagnosis and observation were not performed in those reports [6, 7]. On the other hand, there have been several reports of retinal vascular abnormalities in NF1 [9–12], but there are no reports of pediatric NF1 cases with stenosis of the ophthalmic artery and internal carotid artery simultaneously, with severe retinal vascular abnormalities and ocular ischemic syndrome. Therefore, here we report the diagnosis and clinical course using various imaging modalities in an extremely rare, symptomatic NF1 case with stenosis of both the ophthalmic and internal carotid arteries in a child.

## Case presentation

**Fig. 1:**
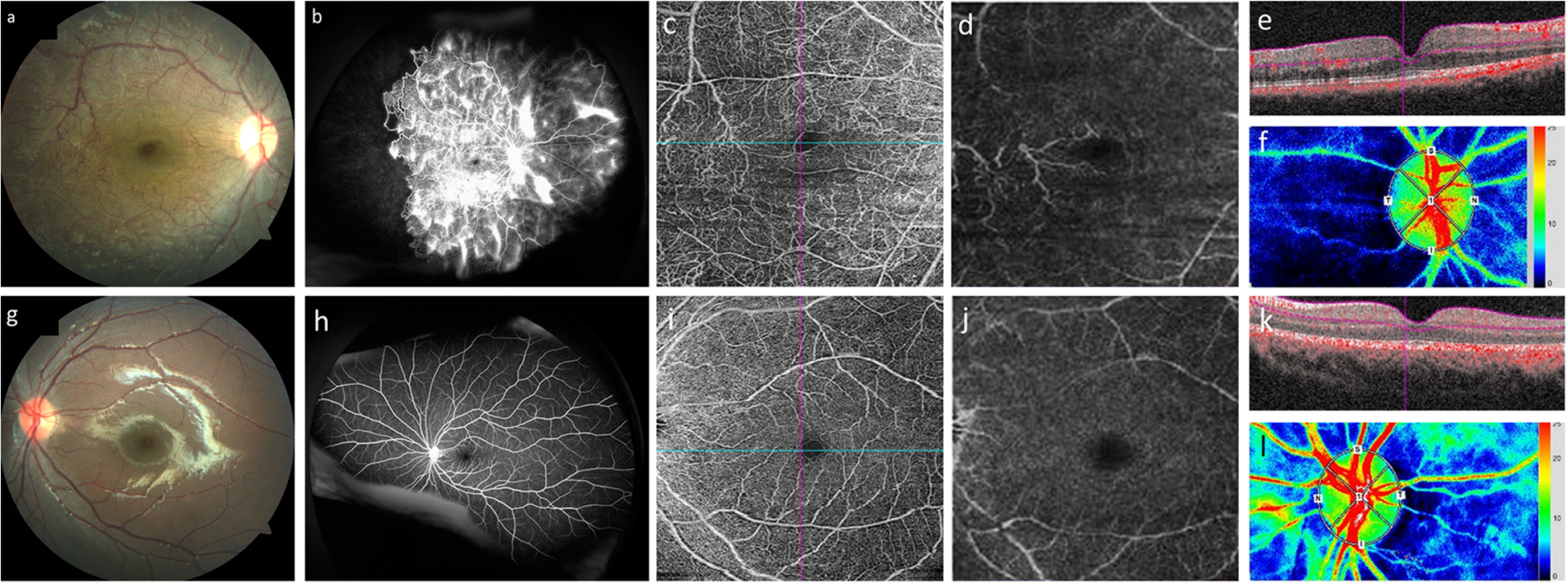
Images from before treatment for ocular ischemic syndrome with retinal vascular abnormalities due to unilateral ocular artery and internal carotid artery stenosis in a pediatric NF1 patient. Color fundus photographs showed poor retinal color and severe retinal vascular abnormalities in the right eye. Vascular abnormalities of the retina formed a complex vascular plexus; some of the abnormalities had corkscrew-like shapes. The foveal reflex had disappeared and optic disc pallor was visible (**a**). Ultra-widefield fluorescein angiography revealed multiple sites of neovascularization, from the posterior pole to the equator, and a large non-perfusion area was observed in the peripheral retina in the right eye (**b**). Optical coherence tomography angiography (OCTA) *en-face* images showed retinal vascular abnormalities in the superficial and deep retinal layers in the right eye (**c**: superficial retinal layer, **d**; deep retinal layer). In OCTA B-scan images, thickening of the inner retina of the right eye was apparent, and there was a slight difference in the retinal surface and deep blood flow signals between the left and right eyes (**e**: right eye, **k**: left eye). Some vascular abnormalities on the temporal side of the macula were also observed as having traffic in the superficial and deep retinal layers (**c**–**e**). Furthermore, deformation of the foveal avascular zone (FAZ) was observed in the right eye and the area of the FAZ was 63.2% larger in the right eye than in the left eye (**c**: right eye, **i**: left eye). Laser speckle flowgraphy (LSFG) imaging showed a marked decrease in chorioretinal blood flow compared with the left eye (**f**: right eye, **l**: left eye). In the left eye, no abnormal findings were observed throughout the course of observation (**g**–**l**)

A 6-year-old girl diagnosed with NF1 presented at our hospital with gradual visual loss in the right eye. Best corrected visual acuity (BCVA) was 20/40 OD and 20/20 OS. Iris Lisch nodules were found in both eyes. The intraocular pressure (IOP) was 12 mmHg in the right eye and 13 mmHg in the left eye. In addition, no iris rubeosis, inflammation or relative afferent pupillary defect was observed in either eye. Severe retinal vascular abnormalities with tortuous-vessel form and optic disc pallor were observed in the right fundus (Fig. 1a). Ultra-widefield fluorescein angiography revealed multiple sites of neovascularization and a large non-perfusion area in the peripheral retina (Fig. 1b). Optical coherence tomography angiography (OCTA) showed vascular abnormalities with changed blood flow signal in the superficial and deep retinal layers in the right eye (Fig. 1c–e). Laser speckle flowgraphy (LSFG) showed that the averaged mean blur rate (MBR) of the right optic nerve head region was 28.7% lower than that of the left eye (MBR values; R = 18.6, L = 26.1), and that the chorioretinal blood flow was substantially decreased in the right eye (Fig. 1f). No abnormal findings were observed in the left eye (Fig. 1g–l).

**Fig. 2:**
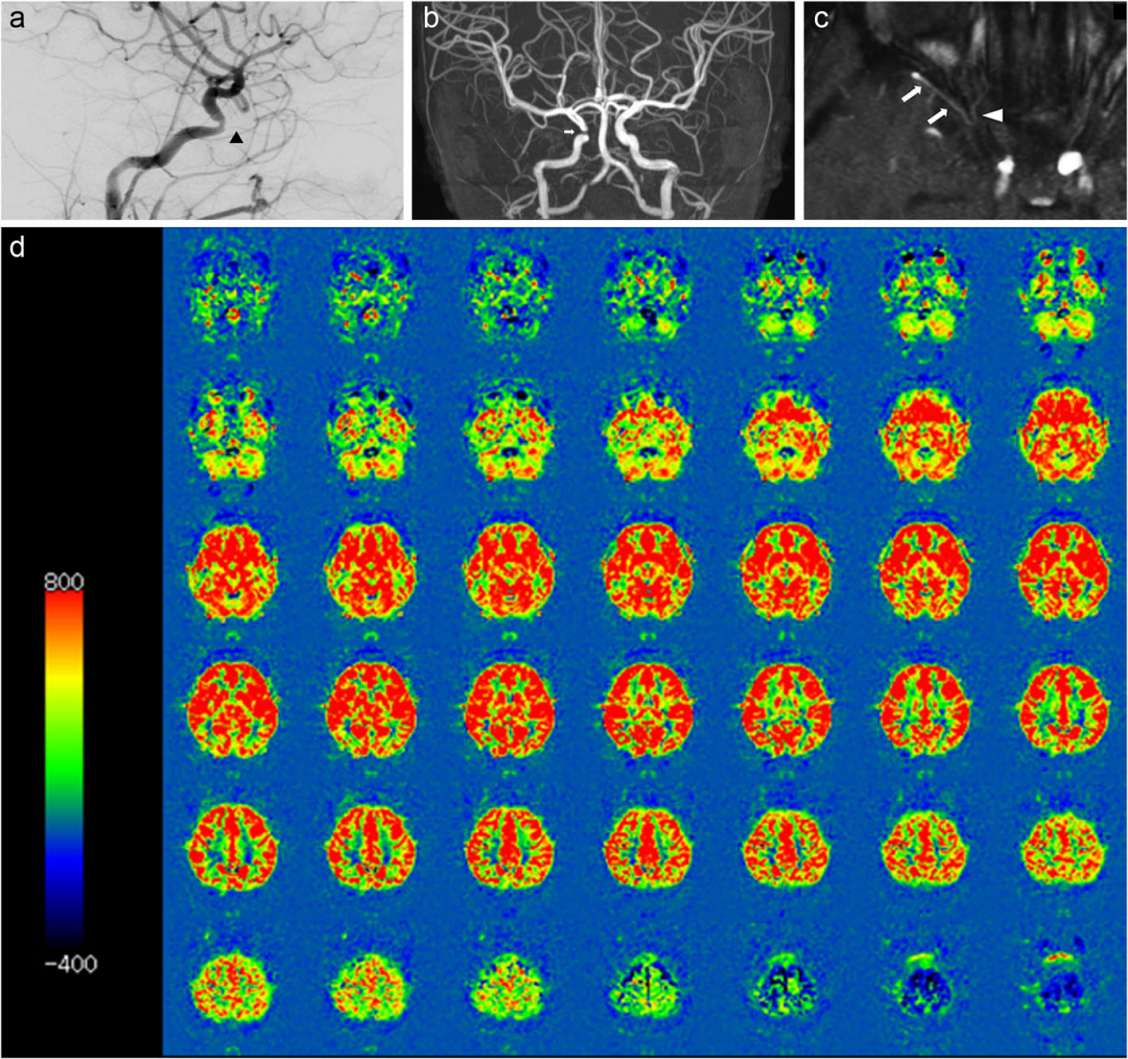
Cerebral angiography, MR angiography (MRA) and arterial spin labeling (ASL) images from MRI. Cerebral angiography revealed significant stenosis of the right eye artery throughout (**a**; arrow head). MRA showed a marked stenosis of the right internal carotid artery at the ophthalmic segment (**b**; arrow). MRA shows the right middle meningeal artery (c; arrows) supplying the orbit via the recurrent meningeal artery (**c**; arrow head). In ASL MRI images, there was no difference in the cerebral blood flow between the left and right sides, nor a decrease in blood flow on the right side (**d**)

Cerebral angiography revealed the right ophthalmic artery was significantly narrowed throughout (Fig. 2a). In addition, Magnetic resonance (MR) angiography revealed that the right internal carotid artery was significantly narrowed in the ophthalmic segment (Fig. 2b). In addition, the patient’s right ophthalmic arteries originated from the middle meningeal artery, not from the internal carotid artery, as they normally do (Fig. 2c). We diagnosed ocular ischemic syndrome with retinal vascular abnormalities resulting from a unilateral ocular artery and internal carotid artery stenosis in NF1.

**Fig. 3:**
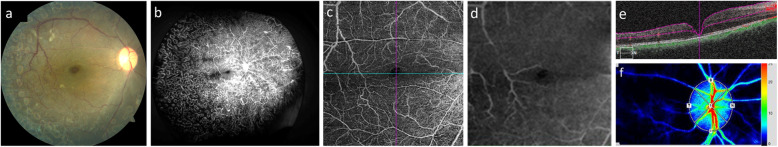
Imaging after a total of two treatments, including panretinal photocoagulation and intravitreal bevacizumab injection. After treatment, the central retinal arterioles and venules on the optic disc had narrowed, compared with before treatment. There was a significant decrease in vascular abnormalities in the surface of the retina after treatment of the right eye (**a**). Regression of retinal neovascularization was visible on fluorescein angiography after treatment (**b**). In OCTA *en-face* and B-scan images, a significant proportion of the vascular abnormalities had disappeared, except for some that remained in the superficial and deep retinal layers after treatment (**c**: *en-face* image of superficial retinal layer, **d**: *en-face* image of deep retinal layer, **e**: B-scan image; these are all images taken after treatment of the right eye.). Chorioretinal blood flow further decreased after treatment (**f**: right eye after treatment)

We examined the treatment while observing for a time. We also examined the possibility of surgical intervention by a neurosurgeon, but concluded that direct intervention on the ophthalmic artery was impossible. After a while, iris rubeosis appeared in the right eye. Since IOP subsequently increased to 35 mmHg and neovascular glaucoma with slight vitreous hemorrhage developed, treatment with panretinal photocoagulation (160–180 mW, total 3241 shots; NIDEK GYC1000, NIDEK CO., LTD., Aichi, Japan) was performed, after intravitreal bevacizumab injection (0.5 mg), twice within 6 months. After 2 treatments, IOP normalized to 10 mmHg, but BCVA decreased to 20/100 OD. A significant portion of the vascular abnormalities disappeared, but some remained in the fundus photograph and OCTA *en-face* images (Fig. 3a, c, d). OCTA B-scan image showed a decrease in blood flow signals in the superficial and deep retinal layers (Fig. 3e). In addition, retinal neovascularization disappeared (Fig. 3b). In LSFG after treatment, the average MBR in the optic nerve head region decreased by 62.7% compared with the left eye (MBR values; R = 9.4, L = 28.7). A marked decrease in chorioretinal blood flow was also confirmed on the LSFG image (Fig. 3f).

Although stenosis of the internal carotid artery was observed, there was no left-right difference or abnormality in cerebral blood flow on arterial spin labeling (Fig. 2d). Therefore, careful follow-up, while taking antiplatelet medication (50 mg of aspirin daily), is currently being conducted with neurosurgeons.

## Discussion and conclusions

NF1 is a hereditary disease that generally causes systemic neurofibroma [1]. In NF1, various complications of systemic vascular stenosis or occlusion that occur in childhood include moyamoya disease and ocular ischemic syndrome [2–5]. However, the cause of vascular stenosis in NF1 is still unclear. So far, there have been only two brief reports of ophthalmic artery occlusion with intracranial lesions that developed in NF1 [6, 7]. There is also a report of unilateral peripheral retinal vascular occlusion in a young woman with NF 1 [8]. No case like this, a child with NF1 who has severe stenosis over the entire length of the ophthalmic artery, has been reported to-date, and this is considered to be an extremely rare pathogenesis. If there is no severe stenosis of the ophthalmic artery, and only stenosis of the internal carotid artery, the flow in the ophthalmic artery is maintained by the regurgitation from the external carotid artery. However, when the ophthalmic artery, itself, is stenotic, it is difficult to secure blood flow by regurgitation. In addition, the pathogenesis is more complicated because the origin of the ophthalmic artery is the middle meningeal artery, not the internal carotid artery, in this case.

In addition, there have been some reports on retinal vascular abnormalities associated with NF1. The characteristic vascular abnormalities have been described as isolated tortuous vessels, corkscrew-like form and moyamoya-like type originating from retinal veins [9–12]. According to these reports, various retinal vascular abnormalities are seen in 6.1–37.5% of NF1 cases [9, 11, 12]. Furthermore, retinal vascular abnormalities with a simple tortuous-vessel form are the most frequent at 73.4%, and their frequency increases with aging [12]. In a large cohort study of 294 NF1 patients, a significant proportion of patients with retinal vascular abnormalities in the superficial layer have an association with vascular abnormalities in the deep layer of the retina [11]. In only 2 out of 18 cases were the retinal vascular abnormalities associated with systemic vascular abnormalities, such as renal artery stenosis. Therefore, in that report, the relationship between retinal vascular abnormalities and systemic vascular stenosis was low [11].

However, in the previous reports [9–12], there were no complications with severe ophthalmic artery stenosis, as in this case, and the degree of retinal vascular abnormalities in this case is extremely severe, as compared with the images in previous reports [9–12]. In general, the detailed condition of ophthalmic artery stenosis can be observed only with cerebral angiography. In particular, cerebral angiography for children needs to be performed under general anesthesia and is particularly invasive, so it is usually not performed unless stenosis of the internal carotid artery or intracranial lesions is present. The cause of retinal vascular abnormalities are unknown [11], but some patients with retinal vascular abnormalities may have ophthalmic arterial abnormalities. There have been reports examining the involvement of neurofibromin as a cause of retinal neovascularization in NF1, and future research results are anticipated [13]. In this case, vascular abnormalities in the surface layer, observed with fundus photograph and OCTA *en-face* images, were partially of the corkscrew-like form. However, they were observed to form a complex vascular plexus, so it is difficult to say that they were isolated tortuous vessels. In addition, retinal vascular abnormalities on the temporal side of the macula, in the deep retinal layer, were observed using OCTA *en-face* images. These were observed from the blood-flow signal findings on the OCTA *en-face* and B-scan images, as if they are partially communicating with the superficial abnormal blood vessels, as reported previously [11]. In this case, the form and extent of the vascular abnormalities are slightly different from those in previous reports [9–12]. In fact, some vascular abnormalities reappeared due to exacerbations. The reason may be that complications of ocular ischemic syndrome due to ophthalmic artery stenosis modify the original vascular abnormalities.

After two treatments, a significant amount of the vascular abnormalities disappeared, but not all. It is unclear whether photocoagulation or the intravitreal bevacizumab injection produced the response because both of these treatments were performed under general anesthesia. The treatments improved the retinal vascular abnormalities and decreased the intraocular pressure, but the visual acuity decreased and the chorioretinal blood flow, as indicated by LSFG, was further reduced in the right eye compared with the preoperative findings. The decrease in the chorioretinal blood flow may be due to the effects of treatments or to progression of the ophthalmic artery stenosis, but the exact cause is unknown. Although the disease settled down with treatment, visual function had, unfortunately, declined.

We have described the diagnosis and treatment of ocular ischemic syndrome with retinal vascular abnormalities due to unilateral ocular artery and internal carotid artery stenosis in a child with NF 1. Various imaging modalities are useful for diagnosing diseases with complicated pathogenesis, such as this case. In addition, these imaging methods make it possible to evaluate the disease state and therapeutic effects.

For the treatment of this condition, direct intervention on the ophthalmic artery would be difficult because it can cause blindness. The biggest problem in this case is the inability of surgical treatment to normalize blood flow in the ophthalmic artery in the right eye. The patient is currently being monitored. In this case, fundamental intervention is impossible and future treatment is expected to be difficult.

## Data Availability

The dataset generated during and/or analyzed during the current study are available from the corresponding author on reasonable request.

## Abbreviations

NF1: Neurofibromatosis type 1
BCVA: Best corrected visual acuity
IOP: Intraocular pressure
OCTA: Optical coherence tomography angiography
LSFG: Laser speckle flowgraphy
MBR: Mean blur rate
ASL: Arterial spin labeling
MR: Magnetic resonance
FAZ: Foveal avascular zone
